# Comparison of second-generation hydrogel embolic coils and bare platinum coils in ruptured intracranial aneurysm treatment: A prospective, multicenter, randomized controlled study

**DOI:** 10.1007/s10143-026-04212-1

**Published:** 2026-03-18

**Authors:** Seung Pil Ban, O-Ki Kwon, Young Deok Kim, Hwan Seok Shim, Seung Bin Sung, Chang Hyeun Kim, Hyoung Soo Byoun

**Affiliations:** 1 Departments of Neurosurgery, Seoul National University Bundang Hospital, 82 Gumi-ro 173 beon-gil, Bundang-gu, Seongnam-si, Gyeonggi-do 13620 Korea; 2 Department of Neurosurgery, Seoul National University College of Medicine, Seoul, Korea; 3 Departments of Neurosurgery, Pusan National University Yangsan Hospital, Pusan National University School of Medicine, Yangsan, Korea; 4 Departments of Neurosurgery, Chungnam National University Sejong Hospital, Sejong, Korea; 5 Department of Neurosurgery, Chungbuk National University Hospital, Chungbuk National University College of Medicine, Cheongju, Korea

**Keywords:** Ruptured aneurysm, Coil embolization, Hydrogel embolic coil, Recurrence

## Abstract

To reduce the recurrence of cerebral aneurysms, second-generation hydrogel embolic coils (HECs) have been developed. We aimed to compare the impacts of bare platinum coils (BPCs) and HECs on the recurrence of ruptured intracranial aneurysms (RIAs). In this study, participants with an RIA were randomly assigned to the BPC or HEC group at a 1:1 ratio. The primary outcome was any recurrence during the 12-month follow-up period. The secondary outcomes included retreatment, procedural complications, delayed cerebral ischemia, favorable clinical outcomes (modified Rankin Scale score ≤ 2), ventriculoperitoneal shunt operation, and the coil’s ease of manipulation. After excluding 11 patients, 57 patients were ultimately included for analysis (BPC, n = 26; HEC, n = 31). The primary outcome was not statistically significant between 2 groups (BPC group: 10/26 [38.5%]; HEC group: 6/31 [19.4%]; P = 0.144). The secondary outcomes did not differ between the 2 groups, except for the coil’s ease of manipulation (P = 0.007). Multivariate analysis revealed no significant difference between the two groups in terms of the primary outcome (rate of recurrence [OR = 0.46; 95% CI, 0.13–1.61; P = 0.223]) or in terms of the secondary outcomes, including retreatment (OR = 0.77; 95% CI, 0.12-5.00; P = 0.766), favorable clinical outcomes (OR = 1.05; 95% CI, 0.16–6.90; P = 0.957), or VP shunt operation (OR = 0.11; 95% CI, 0.01–1.54; P = 0.102). Among patients who underwent coil embolization for an RIA, there were no significant differences between the two groups in terms of the rates of recurrence, retreatment, favorable clinical outcomes or VP shunt operation during the 12-month follow-up period. Given the small sample size, these results should be interpreted with caution. This study is a registered clinical trial. It was prospectively registered in the ClinicalTrials.gov (NCT04988503) on July 23, 2021.

**Supplementary Information:**

The online version contains supplementary material available at 10.1007/s10143-026-04212-1.

## Introduction

Recurrence and the need for retreatment remain significant limitations of endovascular treatment, and these risks are known to be high in cases of ruptured intracranial aneurysms RIAs [1]. Some studies have reported that treatment with flow diverters may reduce the recurrence rate; however, the use of flow diverters for RIAs remains controversial due to concerns about thromboembolic and hemorrhagic complications [2–4]. Accordingly, coil embolization remains an essential modality in the management of RIAs, with ongoing efforts aimed at minimizing recurrence rates. Hydrogel embolic coils (HECs) have been developed as part of these efforts; HECs expand in volume after deployment into the aneurysm, thereby aiming to improve aneurysm healing and durability. A randomized controlled trial (RCT) utilizing first-generation HECs demonstrated a tendency toward lower major recurrence [5]; however, HECs were not widely adopted due to challenges related to device handling. Multiple RCTs have examined the use of second-generation HECs, which are designed with a reduced hydrogel volume to increase the ease of manipulation. The results have revealed that second-generation HECs lead to lower recurrence rates than bare platinum coils (BPCs) in small- to medium-sized aneurysms [6–8].

However, the participants in the previous RCTs were predominantly patients with unruptured intracranial aneurysms (UIAs). Although a subgroup analysis has suggested that HECs may also reduce recurrence in RIAs [9], these findings did not result from a direct comparison between BPCs and HECs in the context of RIAs. Additionally, a previous meta-analysis reported that the recurrence rate after coil embolization was not associated with the type of coil used [10].

Therefore, this prospective RCT aimed to directly compare the effects of BPCs and second-generation HECs on recurrence rates in patients with an RIA – a population that is known to have a higher risk of recurrence following coil embolization.

## Methods

### Study design and participants

This prospective, multicenter, randomized study was conducted across three hospitals. The planned number of participants was 118 (59 patients in each group), and the study period was initially set from August 2021 to July 2023. However, the last patient was randomized in February 2024, and a total of 68 patients were enrolled. The final follow-up was completed in February 2025. This study was designed in accordance with the Consolidated Standards of Reporting Trials (CONSORT) guidelines (Supplementary Method 1), and the full trial protocol is available in the Supplementary Method 2. This trial was approved by the Institutional Review Boards (IRBs) of all the participating institutions (IRB numbers of the institutions: B-2012-652-002, 2021-02-005, and 03-2021-005), and written informed consent was obtained from all the participants before randomization. If the patient was conscious and capable of making an informed decision, the study protocol was explained directly to the patient, and written informed consent was obtained. If the patient had impaired consciousness and was unable to provide consent, written informed consent was obtained from a legally authorized representative approved by the IRB. This study was prospectively registered at ClinicalTrials.gov (NCT04988503).

All consecutive patients aged at least 19 years old who presented to the emergency department, were diagnosed with an RIA, and planned to undergo coil embolization were screened for eligibility. We excluded patients who had an RIA previously treated via endovascular treatment, patients with severe comorbidities or patients with concomitant intracranial lesions. The detailed inclusion and exclusion criteria of this study are described in the Supplementary Method 3.

### Randomization and study procedures

Patients with an RIA were randomly assigned to the BPC or HEC group at a 1:1 ratio via a computer-generated randomization sequence (http://www.randomization.com). In subjects assigned to the HEC group, endovascular treatment was performed using second-generation HECs, including HydroFrame (Microvention/Terumo), HydroSoft 3D (Microvention/Terumo), and HydroSoft Helical (Microvention/Terumo), whenever feasible. When they were deemed necessary, BPCs were used in combination; however, the total length of HECs was required to constitute > 50% of the overall deployed coil length. The use of other types of bioactive coils, such as polyglycolic acid coils (Cerecyte; Cerenovus) and polyglycolic acid/lactide coils (Matrix; Stryker), was not permitted.

All coil embolizations were performed under general anesthesia by experienced operators with more than 5 years of experience. If external ventricular drainage (EVD) catheter insertion was required, it was performed first. In cases where stent deployment was needed, intravenous tirofiban was administered during the procedure and subsequently transitioned to oral antiplatelet therapy after the procedure. In patients who did not undergo EVD insertion but required cerebrospinal fluid (CSF) drainage, lumbar drainage catheter insertion was performed under fluoroscopy following the procedure, and the patients were subsequently managed in the intensive care unit (ICU). The detailed protocol of the coil embolization procedures used in this study is described in the study protocol (Supplementary Method 2). After the procedure, once the patient was stabilized after ICU care, they were transferred to the general ward. After achieving a condition suitable for discharge, the patient was transferred to a rehabilitation hospital or discharged home. All patients in the BPC and HEC groups were regularly followed up on the same schedule until 12 months after coil embolization. The radiological evaluation was repeated at 3 months (X-ray), 6 months (magnetic resonance angiography) and 12-month (digital subtraction angiography [DSA]) after the procedure to determine the changes in the degree of occlusion.

### Outcomes

The primary outcome was any recurrence defined as any progression in the Raymond-Roy occlusion classification class during the 12-month follow-up period [11].

The secondary outcomes were as follows: (1) retreatment during the 12-month follow-up period; (2) procedural complications; (3) delayed cerebral ischemia (DCI); (4) favorable clinical outcomes (modified Ranking Scale [mRS] score ≤ 2) at 12 months after coil embolization; (5) rate of ventriculoperitoneal (VP) shunt operation during the 12-month follow-up period; and (6) ease of coil manipulation. DCI was defined as the occurrence of a new focal neurological deficit or a decrease of at least 2 points in the total score or any individual component of the Glasgow Coma Scale, which was not apparent immediately after aneurysm occlusion and persisted for at least one hour [12]. The ease of coil manipulation is assessed by the operator during the procedure via a numeric scale from 0 to 10 (0 = extremely difficult handling and 10 = extremely easy handling).

The angiographic and clinical outcomes were self-assessed by the treating investigators.

### Statistical analysis

Subgroup analysis in a previous study reported that the rates of recurrence of recently ruptured medium-sized aneurysms in the BPC and second-generation HEC groups were 64.4% and 37.3%, respectively [9]. On the basis of this previous study, we assumed that the recurrence rate would be 64.4% in the BPC group and 37.3% in the HEC group (with a rate difference of 27.1%). The calculated sample size was 106 patients, which provided 80% power to detect this rate difference in the primary outcome at the 2-sided significance level of 0.05. After accounting for a potential 10% dropout rate, we enrolled the study candidates, targeting 118 patients who underwent coil embolization for an RIA (59 in each group). Statistical analysis was performed on the per-protocol population comprising patients who completed the final follow-up according to the assigned protocol.

Baseline demographics are presented as counts and percentages or means and standard deviations (SDs). Comparisons between groups were performed using the Wilcoxon rank sum test and the chi-square test or Fisher’s exact test as appropriate. Multivariate logistic regression analysis was conducted to evaluate the differences in the following outcomes between the BPC and HEC groups: (1) any recurrence during the 12-month follow-up period, (2) retreatment during the 12-month follow-up period, and (3) favorable clinical outcomes at the 12-month follow-up. Variables with *P* < 0.2 in the univariate analyses were included in the multivariate analysis and the model was adjusted for dyslipidemia, dome-to-neck ratio, and the use of a stent after excluding secondary outcomes that could not be clearly categorized as binary outcomes or had low event counts to estimate odds ratios (ORs). The findings are presented as coefficients, *P* values, ORs, and 95% confidence intervals (CIs). Two-sided *P* values less than 0.05 were considered statistically significant. Statistical analyses were performed using IBM SPSS statistics 26.0.

## Results

### Study participants and characteristics

**Fig. 1:**
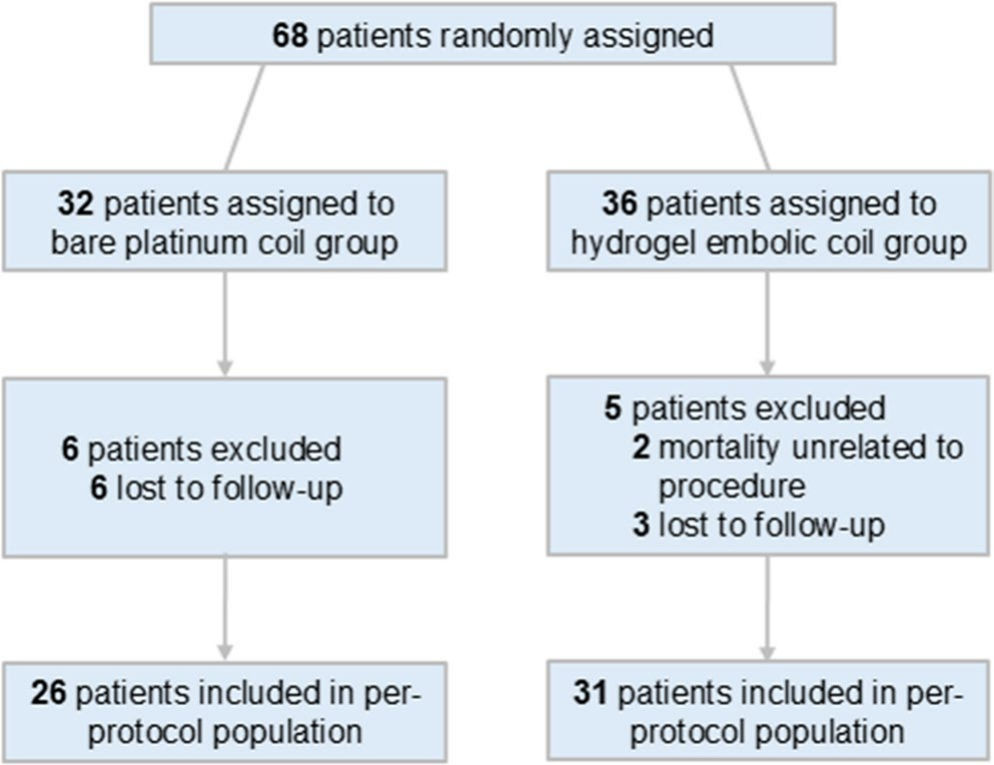
Flow diagram of patient selection

Owing to HEC supply issues at the participating centers and the low enrollment rate among eligible patients, a total of 68 patients with an RIA were enrolled and randomized in this study between August 2021 and February 2024. Eleven patients were excluded due to loss to follow-up (*n* = 9) or death unrelated to the procedure (*n* = 2). Ultimately, a total of 57 patients were included for analysis (Fig. 1).

The baseline characteristics of the 57 included patients are summarized in Table 1.

**Table 1:** Baseline characteristics

Characteristics	Patients, No. (%)			
	Overall (*n* = 57)	BPC (*n* = 26)	HEC (*n* = 31)	*P* value
Age, year, mean (SD)	57.1 (13.1)	58.1 (14.0)	56.2 (12.5)	0.597
Female sex	38 (66.7)	19 (73.1)	19 (61.3)	0.407
Smoking	15 (26.3)	6 (23.1)	9 (29.0)	0.765
Drinking	20 (35.1)	8 (30.8)	12 (38.7)	0.587
Medical history				
Hypertension	19 (33.3)	7 (26.9)	12 (38.7)	0.407
Diabetes	7 (12.3)	2 (7.7)	5 (16.1)	0.436
Dyslipidemia	15 (26.3)	4 (15.4)	11 (35.5)	0.132
Stroke	3 (5.3)	2 (7.7)	1 (3.2)	0.587
Preprocedural findings				
mFisher grade				0.914
1	22 (38.6)	11 (42.3)	11 (35.5)	
2	8 (14.0)	4 (15.4)	4 (12.9)	
3	13 (22.8)	5 (19.2)	8 (25.8)	
4	14 (24.6)	6 (23.1)	8 (25.8)	
Hunt and Hess grade				0.515
1	11 (19.3)	5 (19.2)	6 (19.3)	
2	31 (54.4)	13 (50.0)	18 (58.1)	
3	7 (12.3)	5 (19.2)	2 (6.5)	
4	6 (10.5)	3 (11.6)	3 (9.6)	
5	2 (3.5)	0 (0.0)	2 (6.5)	
Aneurysm data				
Maximum size, mm, mean (SD)	6.1 (2.5)	5.9 (2.2)	6.4 (2.7)	0.434
Neck size, mm, mean (SD)	3.0 (1.2)	2.9 (1.2)	3.2 (1.2)	0.369
Dome-to-neck ratio, mean (SD)	1.5 (0.7)	1.6 (0.9)	1.4 (0.4)	0.194
Location				0.627
ICA	18 (31.6)	6 (23.1)	12 (38.7)	
MCA	7 (12.3)	3 (11.5)	4 (12.9)	
ACA	30 (52.6)	16 (61.5)	14 (45.2)	
Posterior	2 (3.5)	1 (3.9)	1 (3.2)	
Technique				0.031
Non-stent-assisted	48 (84.2)	25 (96.1)	23 (74.2)	
Stent-assisted	9 (15.8)	1 (3.9)	8 (25.8)	

*BPC* bare platinum coil, *HEC* hydrogel embolic coil, *SD* standard deviation, *mFisher* modified Fisher, *ICA* Internal carotid artery, *MCA* middle cerebral artery, *ACA* anterior cerebral artery

The baseline characteristics were well balanced between the BPC and HEC groups, except for a higher frequency of stent-assisted technique in the HEC group (BPC group, *n* = 1 [3.9%]; HEC group, *n* = 8 [25.8%]; *P* = 0.031). Coil embolization was successfully performed in all patients. In non-stent-assisted coiling cases, balloon-assisted coiling was not used. Only the single-microcatheter (*n* = 10 [20.8%]) and multiple-microcatheter (*n* = 38 [79.2%]) techniques were performed. The maximum aneurysm size was 6.1 ± 2.5 mm (range, 2.0–12.4 mm). In the BPC group, all procedures were performed exclusively with BPCs, whereas in the HEC group, HECs accounted for 78.7 ± 16.7% (range, 52–100) of the total implanted coil length.

### Primary and secondary outcomes

Angiographic and clinical outcomes for the BPC and HEC groups are summarized in Table 2 and Supplementary Result 1. In accordance with the study protocol, the final angiographic and clinical follow-up was conducted at 12 months (± 1 month window). In addition, the final imaging assessment was performed using DSA in all patients.

**Table 2:** Angiographic and clinical outcomes of patients

	No. (%)			
	Overall (*n* = 57)	BPC (*n* = 26)	HEC (*n* = 31)	*P* value^*^
Primary outcome				
Initial occlusion classification^†^				0.753
Complete	44 (77.2)	21 (80.8)	23 (74.2)	
Residual neck	13 (22.8)	5 (19.2)	8 (25.8)	
Residual sac	0 (0.0)	0 (0.0)	0 (0.0)	
Final occlusion classification^†^				0.685
Complete	30 (52.6)	13 (50.0)	17 (54.8)	
Residual neck	26 (45.6)	12 (46.2)	14 (45.2)	
Residual sac	1 (1.8)	1 (3.8)	0 (0.0)	
Any recurrence	16 (28.1)	10 (38.5)	6 (19.4)	0.144
Secondary outcome				
Retreatment	6 (10.5)	3 (11.5)	3 (9.7)	> 0.999
Procedural complication	1 (1.8)	0 (0.0)	1 (3.2)	> 0.999
Delayed cerebral ischemia	2 (3.5)	2 (7.7)	0 (0.0)	0.204
Favorable clinical outcome (mRS ≤ 2)	51 (89.5)	23 (88.5)	28 (90.3)	> 0.999
CSF drainage^‡^	28 (49.1)	15 (57.7)	13 (41.9)	0.292
VP shunt operation	5 (8.8)	4 (15.4)	1 (3.2)	0.167
Ease of coil manipulation, mean (SD)	7.5 ± 1.5	8.8 ± 0.5	6.3 ± 1.0	0.007

*BPC* bare platinum coil, *HEC* hydrogel embolic coil, *CSF* cerebrospinal fluid, *VP* ventriculoperitoneal, *mRS* modified Rankin Scale, *SD* standard deviation*The P value measured the comparison between the BPC group and HEC group.^†^Aneurysm obliteration grades were classified as described by the Raymond-Roy occlusion classification.^‡^Cases in which external ventricular drainage catheter insertion or lumbar drainage catheter insertion was performed either before or after the procedure are included.

Complete occlusion was observed in 44 patients (77.2%) after the initial treatment, and at the 12-month follow-up, 30 patients (52.6%) maintained complete occlusion. Sixteen patients (28.1%) experienced deterioration in aneurysm occlusion classification at the 12-month follow-up compared to the initial assessment. Among the patients who experienced recurrence, six (6.5%) underwent retreatment–four with stent-assisted coiling and two with surgical clipping. A thrombus developed in one patient during stent-assisted coiling and was successfully resolved after the intra-arterial administration of tirofiban. No cases of postprocedural rebleeding were observed. A total of 28 patients (49.1%) underwent CSF drainage (12 with EVD and 16 with lumbar drainage), and 5 patients (8.8%) required a VP shunt operation. DCI due to vasospasm occurred in two patients (motor transient ischemic attack, *n* = 1; acute infarction, *n* = 1). During the follow-up period, the mRS score worsened in 2 patients, whereas the remaining 55 patients had either maintained or improved mRS score. One patient died shortly after the procedure due to clinical deterioration caused by rebleeding that occurred prior to treatment, and another patient died from a COVID-19 infection unrelated to the procedure, resulting in a total of two deaths during the follow-up period.

There were no significant differences between the two groups in terms of initial or final aneurysm occlusion classification. During the 12-month follow-up period, recurrence was observed in 10 of 26 patients (38.5%) in the BPC group and in 6 of 31 patients (19.4%) in the HEC group; this difference was not statistically significant (*P* = 0.144).

There were no significant differences between the two groups in terms of secondary outcomes, including retreatment, procedural complications, DCIs, the incidence of VP shunt operation during the 12-month follow-up period, or favorable clinical outcomes. However, operators rated BPCs as significantly easier to handle during coil deployment than HECs (8.8 ± 0.5 vs. 6.3 ± 1.0; *P* = 0.007).

Because 11 patients (16%) were excluded, sensitivity analyses using single imputation were conducted for the primary and secondary outcomes under two assumptions: a ‘worst-case’ scenario, in which all censored cases were imputed as events, and a ‘best-case’ scenario, in which all censored cases were imputed as event-free. The results revealed no substantial differences compared with the outcomes of the original analysis (Supplementary Results 2 and 3).

### Multivariate analysis

The results of the multivariate logistic regression analysis are summarized in Table 3.

**Table 3:** Multivariate logistic regression analysis^*^

Variables	Multivariate^†^	
	Odds ratio (95% CI)	*P* value
Primary outcome		
Any recurrence	0.46 (0.13–1.61)	0.223
Secondary outcome		
Retreatment	0.77 (0.12-5.00)	0.766
Favorable clinical outcome (mRS ≤ 2)	1.05 (0.16–6.90)	0.957
VP shunt operation	0.11 (0.01–1.54)	0.102

*CI* confidence interval, *mRS* modified Rankin Scale^*^Odds of hydrogel embolic coil versus bare platinum coil group.^†^Adjusted for dyslipidemia, dome-to-neck ratio, and use of a stent.

There were no significant differences between the two groups in terms of the primary outcome (rate of recurrence [OR = 0.46; 95% CI, 0.13–1.61; *P* = 0.223]) or in terms of the secondary outcomes, including retreatment (OR = 0.77; 95% CI, 0.12-5.00; *P* = 0.766), favorable clinical outcomes (OR = 1.05; 95% CI, 0.16–6.90; *P* = 0.957), or VP shunt operation (OR = 0.11; 95% CI, 0.01–1.54; *P* = 0.102).

## Discussion

Recurrence is a major concern for patients with RIAs undergoing coil embolization. This recurrence may also be influenced by the type of coil used. In this study, we found no significant differences in the rates of recurrence, retreatment, or favorable outcomes between the BPC and HEC groups during the 12-month follow-up period after coil embolization for an RIA.

After endovascular coil embolization for RIAs, 11% of patients required retreatment and 2.1% experienced rebleeding [13]. In particular, the incidence of rerupture was higher in cases of residual aneurysm with contrast filling along the aneurysm wall than in cases of simple neck recanalization [14]. However, recurrence can precede rupture, even in aneurysms initially considered completely occluded [13]. Therefore, in RIAs, efforts to reduce recurrence are essential to minimize the risk of rebleeding and to decrease the potential procedural complications associated with retreatment. To prevent the recurrence of coil aneurysms, bioactive coils such as first-generation HECs, Matrix, and Cerecyte were introduced with the aim of increasing durability by promoting stable thrombus formation and facilitating neoendothelialization. However, previous RCTs have failed to demonstrate the efficacy of these early-generation bioactive coils over BPCs [5, 15, 16]. Nevertheless, a study on HECs revealed a trend toward reduced major recurrence compared with BPCs, thereby generating interest in the use of HECs. However, first-generation HECs have limitations due to the large amount of hydrogel used, which results in increased coil stiffness and time constraints for placement. To overcome these limitations, second-generation HECs were developed. In two previous RCTs using second-generation HECs, the HEC group exhibited better outcomes than the BPC group [6, 8]. However, both studies included a combined population of UIAs and RIAs, with the majority of patients having UIAs; moreover, one study specifically reported that the effect of second-generation HEC seemed more pronounced in UIAs [8]. Furthermore, although patients with UIAs and those with RIAs were combined in the analysis, a recently published RCT also reported no significant difference in the recanalization rate between the BPC and HEC groups [7]. Because the results regarding the efficacy of HECs remain inconsistent and since they are based on a combined population of UIAs and RIAs, there are limitations in directly applying these findings to the treatment of RIAs. In this study, which focused exclusively on RIAs, no difference in the rate of any recurrence was observed between the BPC and HEC groups. This finding may be attributed to the higher rate of recurrence among RIAs than among UIAs, suggesting that the tendency for recurrence is similar regardless of the type of coil used. Another possible explanation is that the criteria used to define the HEC group in this study might have influenced the results. A study that used a threshold of 90% for HECs reported a significantly lower recurrence rate in the HEC group [6], whereas a study using a 50% threshold reported no significant difference [7]. In the present study, the HEC group was defined as cases in which HEC coils accounted for more than 50% of the total coils used rather than cases in which HECs were used exclusively. Owing to this classification approach, the range and median proportion of mixed use of BPC in the HEC group in this study may have influenced the evaluation of the efficacy of pure HEC and may have contributed to the absence of a significant difference in recurrence rates between the two groups. Another possible reason is that, despite extending the study period by seven months, the originally planned sample size of 118 patients was not achieved because of supply issues at participating centers and a low enrollment rate among eligible patients. This might have led to the reduced statistical power in this study. In fact, given the observed effect size and the existing sample size in this study, a post hoc power analysis demonstrated an estimated power of approximately 42%, suggesting that the study may have been underpowered. However, as one of the prospective RCTs specifically targeting RIAs, this study directly compares second-generation HEC with BPC, and its negative results have important reference value for clinical decision-making in this specific population. If validated in a large-scale trial focusing on RIAs in the future, these results could influence clinical practice guidelines.

In terms of the relationship between the maximum aneurysm size and the risk of recurrence, according to a previous study, HECs appear to be effective in preventing the recurrence of medium-sized aneurysms (range 3–14 mm) regardless of their rupture status, aneurysm neck size and procedural angiographic occlusion [6]. However, in a study involving aneurysms measuring 7–20 mm, the recurrence prevention effect of HECs was not demonstrated [7]. In this study, the treated RIAs were medium-sized, with an average diameter of 6.1 mm (range, 2.0-12.4); however, there was no difference in recurrence rates between the BPC and HEC groups. This result suggests that in RIAs, regardless of size, the choice of coil may not influence the risk of recurrence.

As in previous studies [6, 7], there was no significant difference in favorable clinical outcomes between the two groups in this study. The type of coil was found to have no significant effect on clinical outcomes. Although no specific selection criteria were applied other than the presence of an RIA, the majority of the enrolled patients exhibited relatively good clinical status. This may be attributable to our healthcare environment, where patients experiencing acute neurological symptoms are able to access emergency medical services or present directly to the emergency department without significant delay. Therefore, our cohort may have included a lower proportion of poor-grade subarachnoid hemorrhage (SAH) patients. If the study had included a greater number of poor-grade SAH patients, the overall clinical outcomes could be different. This potential limitation should be considered when interpreting the generalizability of our findings. In some studies using first-generation HECs, HEC has been reported to be related to aseptic meningitis and delayed hydrocephalus [17, 18]. We analyzed the rate of VP shunt operation during the follow-up period, and there was no significant difference between the 2 groups. Unlike first-generation HECs, which contain more hydrogel, second-generation HECs are not significantly associated with the occurrence of hydrocephalus.

In coil manipulation, second-generation HECs were designed to be as user friendly as BPCs due to the insertion of a hydrogel in a filament form inside the BPC, leading to delayed gel expansion. Additionally, 1-mm-diameter HECs were added to improve the ease of handling of HECs. Although previous studies have described improvements in coil handling, these claims were not substantiated by formal evaluation of the operator’s subjective experience. In this study, we assessed the perceived ease of coil manipulation among operators and found that HECs were still considered more difficult to handle than BPCs (6.3 vs. 8.6). Although baseline characteristics were similar between the two groups, stent-assisted coiling was more frequent in the HEC group, which may reflect the relatively greater difficulty in manipulating HECs during the procedure. The findings that BPCs were perceived as easier to manipulate is clinically meaningful. Improved handling may shorten the learning curve, particularly for less-experienced surgeons, and may reduce repeated adjustments during deployment, potentially leading to shorter procedural times. This aspect may have practical implications in the setting of RIAs, where time pressure and psychological stress are substantial, and procedural simplicity may help mitigate surgeon burden and enhance procedural stability. However, although the assessment of coil manipulation was performed by neurointerventionists with more than five years of experience, it was based on the surgeons’ subjective perceptions rather than objective quantitative metrics. In addition, blinding to the type of coil was not feasible, which may have introduced potential bias in the evaluation.

This study has several limitations. First, although the multivariate analysis revealed that HEC had no effect on preventing recurrence in patients with an RIA, the main limitation of this study is that it is underpowered (64%) because of an insufficient sample size, having reached only 57.6% of the planned sample size for the reasons discussed earlier. Owing to the insufficient sample size and limited statistical power, the possibility of a false-negative result cannot be excluded. Although a true difference between the HEC and BPC groups may exist, the present study may have failed to detect such a difference. Therefore, the findings should be interpreted with caution. Additional studies with larger sample sizes are needed to validate the study results. Second, to ensure procedural convenience and safety, the HEC group was not defined as a group treated with 100% HEC, which may have limited the ability to comprehensively assess the true effect of HEC. Third, the primary and secondary outcomes of this study were self-assessed by the treating investigators. The radiological outcomes were not assessed by an independent, blinded core laboratory, and the clinical outcomes were not evaluated by an independent clinical events committee. Therefore, the possibility of potential bias in outcome assessment cannot be ruled out. Finally, the 12-month follow-up period is relatively short compared with other studies, thus limiting the ability to assess long-term outcomes on the basis of the results of this study.

## Conclusions

In this study, among patients who underwent coil embolization for an RIA, there was no significant difference between the BPC and HEC groups in terms of the rates of recurrence, retreatment, favorable clinical outcomes, or VP shunt operation during the 12-month follow-up period. Given the insufficient sample size, these results should be interpreted with caution. Further large-scale, adequately powered studies are warranted to clarify the potential role of HECs in reducing recurrence after coil embolization of RIAs.

## Supplementary Information

Below is the link to the electronic supplementary material.

Supplementary Material 1 ( DOCX 235 KB)

## Data Availability

The datasets generated during and/or analyzed during the current study are available from the corresponding author on reasonable request.
