# Supplementary material for: Comparison of second-generation hydrogel embolic coils and bare platinum coils in ruptured intracranial aneurysm treatment: A prospective, multicenter, randomized controlled study
Source: Neurosurg Rev. 2026 Mar 18;49(1):289. doi: 10.1007/s10143-026-04212-1 (PMC12995996; doi:10.1007/s10143-026-04212-1)
Supplement: Supplementary file 1 — Supplementary Material 1 ( DOCX 235 KB) [file 10143_2026_4212_MOESM1_ESM.docx]

**Supplementary information**

Comparison of second-generation hydrogel embolic coils and bare platinum coils in ruptured intracranial aneurysm treatment: A prospective, multicenter, randomized controlled study

**Supplementary Method 1** CONSORT checklist

|  | Section/topic | No | CONSORT 2025 checklist item description | Reported on page no. |
| --- | --- | --- | --- | --- |
|  | **Title and abstract** | | |  |
|  | Title and structured abstract | 1a | Identification as a randomised trial | Title page |
|  |  | 1b | Structured summary of the trial design, methods, results, and conclusions | 3 |
|  | **Open science** | | |  |
|  | Trial registration | 2 | Name of trial registry, identifying number (with URL) and date of registration | 3 |
|  | Protocol and statistical analysis plan | 3 | Where the trial protocol and statistical analysis plan can be accessed | Suppl. Method 2 |
|  | Data sharing | 4 | Where and how the individual de-identified participant data (including data dictionary), statistical code and any other materials can be accessed | Title page |
|  | Funding and conflicts of interest | 5a | Sources of funding and other support (eg, supply of drugs), and role of funders in the design, conduct, analysis and reporting of the trial | Title page |
|  |  | 5b | Financial and other conflicts of interest of the manuscript authors | Title page |
|  | **Introduction** | | |  |
|  | Background and rationale | 6 | Scientific background and rationale | 4 |
|  | Objectives | 7 | Specific objectives related to benefits and harms | 4 |
|  | **Methods** | | |  |
|  | Patient and public involvement | 8 | Details of patient or public involvement in the design, conduct and reporting of the trial | 4-5 |
|  | Trial design | 9 | Description of trial design including type of trial (eg, parallel group, crossover), allocation ratio, and framework (eg, superiority, equivalence, non-inferiority, exploratory) | 4-5 |
|  | Changes to trial protocol | 10 | Important changes to the trial after it commenced including any outcomes or analyses that were not prespecified, with reason | NA |
|  | Trial setting | 11 | Settings (eg, community, hospital) and locations (eg, countries, sites) where the trial was conducted | 4-5 |
|  | Eligibility criteria | 12a | Eligibility criteria for participants | 5 & Suppl. Method 3 |
|  |  | 12b | If applicable, eligibility criteria for sites and for individuals delivering the interventions (eg, surgeons, physiotherapists) | NA |
|  | Intervention and comparator | 13 | Intervention and comparator with sufficient details to allow replication. If relevant, where additional materials describing the intervention and comparator (eg, intervention manual) can be accessed | 5-6 |
|  | Outcomes | 14 | Prespecified primary and secondary outcomes, including the specific measurement variable (eg, systolic blood pressure), analysis metric (eg, change from baseline, final value, time to event), method of aggregation (eg, median, proportion), and time point for each outcome | 6 |
|  | Harms | 15 | How harms were defined and assessed (eg, systematically, non-systematically) | NA |
|  | Sample size | 16a | How sample size was determined, including all assumptions supporting the sample size calculation | 6-7 |
|  |  | 16b | Explanation of any interim analyses and stopping guidelines | NA |
|  | Randomisation: |  |  |  |
|  | Sequence generation | 17a | Who generated the random allocation sequence and the method used | 4 |
|  |  | 17b | Type of randomisation and details of any restriction (eg, stratification, blocking and block size) | 4-5 |
|  | Allocation concealment mechanism | 18 | Mechanism used to implement the random allocation sequence (eg, central computer/telephone; sequentially numbered, opaque, sealed containers), describing any steps to conceal the sequence until interventions were assigned | 5 |
|  | Implementation | 19 | Whether the personnel who enrolled and those who assigned participants to the interventions had access to the random allocation sequence | NA |
|  | Blinding | 20a | Who was blinded after assignment to interventions (eg, participants, care providers, outcome assessors, data analysts) | NA |
|  |  | 20b | If blinded, how blinding was achieved and description of the similarity of interventions | NA |
|  | Statistical methods | 21a | Statistical methods used to compare groups for primary and secondary outcomes, including harms | 6-7 |
|  |  | 21b | Definition of who is included in each analysis (eg, all randomised participants), and in which group | 6-7 |
|  |  | 21c | How missing data were handled in the analysis | 6-7 |
|  |  | 21d | Methods for any additional analyses (eg, subgroup and sensitivity analyses), distinguishing prespecified from post hoc | NA |
|  | **Results** | | |  |
|  | Participant flow, including flow diagram | 22a | For each group, the numbers of participants who were randomly assigned, received intended intervention, and were analysed for the primary outcome | 7 & fig. 1 |
|  |  | 22b | For each group, losses and exclusions after randomisation, together with reasons | 7 |
|  | Recruitment | 23a | Dates defining the periods of recruitment and follow-up for outcomes of benefits and harms | 7 |
|  |  | 23b | If relevant, why the trial ended or was stopped | NA |
|  | Intervention and comparator delivery | 24a | Intervention and comparator as they were actually administered (eg, where appropriate, who delivered the intervention/comparator, how participants adhered, whether they were delivered as intended (fidelity)) | 7-14 |
|  |  | 24b | Concomitant care received during the trial for each group | NA |
|  | Baseline data | 25 | A table showing baseline demographic and clinical characteristics for each group | Table 1 |
|  | Numbers analysed,  outcomes and estimation | 26 | For each primary and secondary outcome, by group:  ● the number of participants included in the analysis  ● the number of participants with available data at the outcome time point  ● result for each group, and the estimated effect size and its precision (such as 95% confidence interval)  ● for binary outcomes, presentation of both absolute and relative effect size | 10-14 |
|  | Harms | 27 | All harms or unintended events in each group | NA |
|  | Ancillary analyses | 28 | Any other analyses performed, including subgroup and sensitivity analyses, distinguishing pre-specified from post hoc | NA |
|  | **Discussion** | | |  |
|  | Interpretation | 29 | Interpretation consistent with results, balancing benefits and harms, and considering other relevant evidence | 14-17 |
|  | Limitations | 30 | Trial limitations, addressing sources of potential bias, imprecision, generalisability, and, if relevant, multiplicity of analyses | 16-17 |

Citation: Hopewell S, Chan AW, Collins GS, Hróbjartsson A, Moher D, Schulz KF, et al. CONSORT 2025 Statement: updated guideline for reporting randomised trials. BMJ. 2025; 388:e081123. <https://dx.doi.org/10.1136/bmj-2024-081123>
© 2025 Hopewell et al. This is an Open Access article distributed under the terms of the Creative Commons Attribution License (<https://creativecommons.org/licenses/by/4.0/>), which permits unrestricted use, distribution, and reproduction in any medium, provided the original work is properly cited.

*We strongly recommend reading this statement in conjunction with the CONSORT 2025 Explanation and Elaboration and/or the CONSORT 2025 Expanded Checklist for important clarifications on all the items. We also recommend reading relevant CONSORT extensions. See [www.consort-spirit.org](http://www.consort-spirit.org).

**Supplementary Method 2** Study protocol

**Comparison of second-generation hydrogel embolic coils versus bare platinum coils in ruptured intracranial aneurysm treatment: a prospective, multicenter, randomized controlled study**

**Principle Investigator:**

O-Ki Kwon, MD, PhD^1,2^

Professor,

Department of Neurosurgery,

Seoul National University Hospital,

82 Gumi-ro 173 beon-gil, Bundang-gu

Seongnam-si, Gyeonggi-do, 13620, Korea

Phone: 82-31-787-7163, Fax: 82-31-787-4097

E–mail: meurokwonoki@gmail.com

**Co-investigator:**

Seung Pil Ban, MD, PhD^1,2^

Young Deok Kim, MD^1^

Hwan Seok Shim, MD^1^

Seung Bin Sung, MD^1^

Chang Hyeun Kim, MD^3^

Hyoung Soo Byoun, MD, PhD^4^

*^1^Departments of Neurosurgery, Seoul National University Bundang Hospital, Seongnam-si, Gyeonggi-do, Korea*

*^2^Department of Neurosurgery, Seoul National University College of Medicine, Seoul, Korea*

*^3^Departments of Neurosurgery, Pusan National University Yangsan Hospital, Pusan National University School of Medicine, Yangsan, Korea*

*^4^Departments of Neurosurgery, Chungnam National University Sejong Hospital, Chungnam National University College of Medicine, Sejong, Korea*

**Funded by:** SNUBH research fund (grant no. 02-2018-0021)

**Registered:** ClinicalTrials.gov Identifier: NCT04988503 / July 23, 2021

**Current Protocol Version:** 2.0 (Feb 04, 2025)

**List of Abbreviation**

BPC Bare platinum coil

CSF Cerebrospinal fluid

EVD Extraventricular drainage

HEC Hydrogel embolic coil

mRS modified Rankin Scale

RIA Ruptured intracranial aneurysm

UIA Unruptured intracranial aneurysm

**Synopsis**

| Title | Comparison of second-generation hydrogel embolic coils versus bare platinum coils in ruptured intracranial aneurysm treatment: a prospective, multicenter, randomized controlled study |
| --- | --- |
| Purpose | In the case of coil embolization for ruptured intracranial aneurysms, recurrence after treatment are highly observed. For its prevention, hydrogel embolic coils have been used. This study aims to analyze the differences in recurrence rates after coil embolization for ruptured cerebral aneurysms based on the type of coil used (bare platinum coils versus hydrogel embolic coils). |
| Design | Prospective, randomized, open-labeled, comparative trial |
| Study period | Three years after institutional review board approval |
| Patient enrollment | Calculated size: 106 (bare platinum coil group, 53; hydrogel embolic coil group, 53)  Target size: 118, considering a dropout rate 10% (bare platinum coil group, 59; hydrogel embolic coil group, 59) |
| Study candidate | Patients with a rupture intracranial aneurysm |
| Study drugs | Not applicable |
| Study devices | Bare platinum coils and hydrogel embolic coils |
| Methods | Study participants diagnosed with ruptured intracranial aneurysms and treated with coil embolization will be divided as follows:  a. Group 1 – Coil embolization using bare platinum coils (defined as cases in which bare platinum coils account for more than 50% of the total length of coils used)  b. Group 2 – Coil embolization using second-generation hydrogel embolic coils (defined as cases in which hydrogel embolic coils account for more than 50% of the total length of coils used) |
| Inclusion criteria | * Patients with an acutely ruptured intracranial aneurysm who plan to undergo coil embolization  * Patient aged over 19 years  * Patient who agrees to this study (with informed consent) |
| Exclusion criteria | * Patient with an allergic reaction to antiplatelets (tirofiban, aspirin, clopidogrel, prasugrel and cilostazol) or contrast  * Patient with a high risk of hemorrhage such intracerebral hemorrhage or severe gastric ulceration  * Patient with a high risk of strokes (over 50% cerebral artery stenosis or cerebral artery occlusion, moyamoya disease, atrial fibrillation)  * Patient with coagulopathy  * Patient with thrombocytopenia (thrombocytopenia (<100,000/mm^3^)  * Patient with liver diseases (>100IU/dL of aspartate aminotransferase or alanine aminotransferase)  * Patient with renal diseases (>2 mg/dL of serum creatinine)  * Patient with uncontrolled congestive heart failure or angina  * Patient with malignant tumors  * Pregnant or breast-feeding patient  * Patient who are determined to be disqualified by researchers |
| Primary endpoint | Any recurrence rate during 12 months follow-up period after coil embolization |
| Secondary endpoint | * Rate of retreatment during 12-month follow-up period  * Periprocedural complication  * Delayed ischemic symptoms  * Good clinical outcome (modified Rankin Scale score ≤ 2) at 12-month after coil embolization  * Rate of ventriculoperitoneal shunt operation during 12-month follow-up period |
| Evaluation schedule | Neurological status: at admission, from coiling to discharge to home, and 1 month, 3 months, 6 months and 12months after coil embolization  Visiting schedule: 1 month, 3 months, 6 months and 12months after coil embolization  Image follow-up: skull X ray at 3-month follow-up, MRA at 6-month follow-up, and 12-month follow-up transfemoral cerebral angiography |
| Statistical analysis | Principle analyses are comparison of primary and secondary outcomes between the bare platinum coil group and hydrogel embolic coil group. All means are presented with their corresponding standard deviations. Continuous data were compared using independent t-tests, while categori­cal variables were compared using the Chi-square test or Fisher’s exact test. Multivariate logistic regression analyses were performed to determine whether differences between the bare platinum coil group and hydrogel embolic coil group. |
| Sponsor | Seoul National University Bundang Hospital Research Fund (02-2018-0021) |

**1. Study Title**

Comparison of second-generation hydrogel embolic coils versus bare platinum coils in ruptured intracranial aneurysm treatment: a prospective, multicenter, randomized controlled study

**2. Study Institute and Location**

1. Seoul National University Bundang Hospital

82 Gumi-ro 173 beon-gil, Bundang-gu, Seongnam-si, Gyeonggi-do, 13620, Korea

2. Seoul National University College of Medicine

103 Daehak-ro, Jongno-gu, Seoul, 03080, Korea

3. Pusan National University Yangsan Hospital

20, Geumo-ro, Mulgeum-eup, Yangsan-si, Gyeongsangnam-do, 50612, Korea

4. Chungnam National University Sejong Hospital

20, Bodeum 7-ro, Sejong-si, 30099, Korea

**3. Investigators**

|  | Name | Department | Position |
| --- | --- | --- | --- |
| Principle investigator | O-Ki Kwon | Neurosurgery^1,2^ | Professor |
| Co-investigator | Seung Pil Ban | Neurosurgery^1,2^ | Associate professor |
| Co-investigator | Young Deok Kim | Neurosurgery^1^ | Assistant professor |
| Co-investigator | Hwan Seok Shim | Neurosurgery^1^ | Assistant professor |
| Co-investigator | Seung Bin Sung | Neurosurgery^1^ | Assistant professor |
| Co-investigator | Chang Hyeun Kim | Neurosurgery^3^ | Associate professor |
| Co-investigator | Hyoung Soo Byoun | Neurosurgery^4^ | Associate professor |

**4. Source of Funding**

SNUBH research fund (grant no. 02-2018-0021)

**5. Background and Purpose**

**5.1. Background**

Endovascular treatment for cerebral aneurysms has been more widely used since the International Subarachnoid Aneurysm Trial.^1^ Nevertheless, recurrence observed after coil embolization remains a persistent concern, and the risk of recurrence has been reported to be higher in ruptured intracrainal aneurysms (RIAs) compared to unruptured intracranial aneurysms (UIAs) (unruptured versus ruptured; 9.2% versus 19.7%).^2^

As a way to overcome this issue, hydrogel embolic coils (HECs)—designed to expand over time in the bloodstream—have been proposed to maintain a high packing density within the cerebral aneurysm during embolization. Several randomized controlled trials (RCTs) have investigated the use of hydrogel embolic coils in the treatment of cerebral aneurysms, and the results have shown that the risk of recurrence is lower with HEC compared to bare platinum coils (BPCs).^3-6^ However, these RCTs included both unruptured and ruptured aneurysms, with the majority of treated cases being unruptured aneurysms. Since the recurrence rate after coil embolization is known to be higher in ruptured intracranial aneurysms compared to unruptured ones, there are limitations in directly applying the findings of previous studies—most of which included predominantly unruptured aneurysms—to ruptured aneurysms.^7^ Additionally, other meta-analyses have reported that the type of coil did not significantly affect the risk of recurrence.^8^ To our knowledge, there have been no prospective and randomized controlled trials directly comparing BPC treatment and HEC treatment for patients undergoing coil embolization of a ruptured intracranial aneurysm (RIA).

**5.2. Purpose**

In a subgroup analysis of the HydroCoil Endovascular Aneurysm Occlusion and Packing Study (HELPS) trials, HECs were associated with statistically significant and clinically relevant lower rates of major recurrence for recently ruptured, medium-sized aneurysms in the HELPS trial.^9^ However, the recurrence rates following treatment with BPC and HEC in RIAs have not been clearly established through direct comparison. Therefore, we aimed to directly compare BPC and HEC in patients with RIAs to investigate differences in recurrence rates and clinical outcomes between the two treatment methods.

**6. Research Materials**

**6.1. Bare platinum coil (BPC) Group**

Bare platinum coils

- Target (Stryker Neurovascular)

- MicroPlex (MicroVention/Terumo)

- Axium (Medtronic)

: except surface-modified coils such as Matrix (Stryker) and Cerecyte (Cerenovus)

**6.2. Hydrogel embolic coil (HEC) Group**

Hydrogel embolic coils

- HydroSoft 3D & Helical (Microvention/Terumo, Tustin, CA, USA)

- HydroFrame (Microvention/Terumo, Tustin, CA, USA)

: the proportion of HEC among the total length used during coil embolization must be at least 50% (a HEC length ≥ 50% of the length of all the coils)

**6.3. Routine protocol of coil embolization procedures for a ruptured intracranial aneurysm (RIA)**

**6.3.1. Non-stent-assisted coil embolization**

If external ventricular drainage (EVD) catheter insertion is required, it should be performed prior to coil embolization. All endovascular procedures will be performed under general anesthesia using an IFNX-8000V (Toshiba Medical System Corporation, Otawara, Tochigi). 7 French (Fr) sheath will be inserted into right common femoral artery. A 7Fr or 6Fr guiding catheter (GuiderSoftip; Stryker Neurovascular, Kalamazoo, MI or Envoy; Cerenovus, Raynham, MA) will be placed at proximal cervical ICA or VA. First, a target aneurysm will be selected using a microcatheter (Excelsior SL-10; Stryker Neurovascular, Kalamazoo, MI). If the procedure can be performed using a single microcatheter, it should be done accordingly; otherwise, treatment should be conducted using either the multiple microcatheter technique or the balloon-assisted technique. Then, the aneurysm will be coiled using BPCs or HECs until complete occlusion is achieved or further coiling is deemed unsafe. Following coil embolization, the coiling microcatheter will be gently removed from the aneurysm. Digital subtraction angiography will be performed to assess the aneurysm occlusion rate and identify any complications. If cerebrospinal fluid (CSF) drainage is required after the procedure, a lumbar drainage catheter is inserted under fluoroscopic guidance. After the coil embolization procedure, the patients are observed in the intensive care unit (ICU) until clinical stabilization is achieved, after which they are transferred to a general ward. After achieving a condition suitable for discharge, the patient is transferred to a rehabilitation hospital or discharged home. All patients in the BPC and HEC groups were regularly followed up on the same schedule until 12 months after coil embolization. The patients’ clinical status was evaluated at 1, 3, 6 and 12 months after the procedure. We instructed the patients to visit the emergency department, regardless of their follow-up schedule, when any event associated with this study occurred, even if it was mild or transient.

**6.3.2. Stent-assisted coil embolization**

If EVD catheter insertion is required, it should be performed prior to coil embolization. All endovascular procedures will be performed under general anesthesia using an IFNX-8000V (Toshiba Medical System Corporation, Otawara, Tochigi). 7 French (Fr) sheath will be inserted into right common femoral artery. If patients were planned to undergo stent-assisted coil embolization for RIAs, tirofiban (glycoprotein IIb/IIIa receptor antagonist) was used of antiplatelet therapy. After insertion of femoral artery sheath, tirofiban was administered intravenously as a continuous infusion of 0.4 ㎍ per kilogram of body weight per minute for 30 minutes, followed by a maintenance dose of 0.1 ㎍ per kilogram per minute. A 7Fr or 6Fr guiding catheter (GuiderSoftip; Stryker Neurovascular, Kalamazoo, MI or Envoy; Cerenovus, Raynham, MA) will be placed at proximal cervical ICA or VA. First, a target aneurysm will be selected using a microcatheter (Excelsior SL-10; Stryker Neurovascular, Kalamazoo, MI). If the procedure can be performed using a single microcatheter, it should be done accordingly; otherwise, treatment should be conducted using either the multiple microcatheter technique or the balloon-assisted technique. Then, the aneurysm will be coiled using BPCs or HECs until complete occlusion is achieved or further coiling is deemed unsafe. Following coil embolization, the coiling microcatheter will be gently removed from the aneurysm. Digital subtraction angiography will be performed to assess the aneurysm occlusion rate and identify any complications. If CSF drainage is required after the procedure, a lumbar drainage catheter is inserted under fluoroscopic guidance. After the coil embolization procedure, the patients are observed in the ICU. Upon arrival at the ICU, dual antiplatelet therapy (100mg aspirin + 20mg prasugrel) is initiated, IV tirofiban is discontinued and the patient is subsequently maintained on 100mg aspirin and 5mg prasugrel. Once the patient is stabilized after ICU care, they are transferred to the general ward. After achieving a condition suitable for discharge, the patient is transferred to a rehabilitation hospital or discharged home. All patients in the BPC and HEC groups were regularly followed up on the same schedule until 12 months after coil embolization. The patients’ clinical status was evaluated at 1, 3, 6 and 12 months after the procedure. We instructed the patients to visit the emergency department, regardless of their follow-up schedule, when any event associated with this study occurred, even if it was mild or transient.

**6.4. Interventional Product**

| **Device name** | **Manufacturer** | **Description** |
| --- | --- | --- |
| Enterprise stent | Cerenovus | Stent |
| Low-profile Visualized Intraluminal Support (LVIS) stent | MicroVention/Terumo | Stent |
| LVIS Jr. stent | MicroVention/Terumo | Stent |
| Neuroform Atlas stent | Stryker Neurovascular | Stent |
| Target detachable coils | Stryker Neurovascular | BPC |
| MicroPlex | MicroVention/Terumo | BPC |
| Axium | Medtronic | BPC |
| HydroSoft (3D & Helical) | MicroVention/Terumo | HEC |
| HydroFrame | MicroVention/Terumo | HEC |

**7. Study population**

118 patients undergoing coil embolization for an RIA

**8. Study Period**

Three years after Institutional Review Board (IRB) approves the study protocol

**9. Eligible criteria, Sample size calculation**

**9.1. Inclusion criteria**

- Patients with an RIA who plan to undergo coil embolization

- Patient aged ≥19 years

- Patient who agrees to this study (with informed consent)

**9.2. Exclusion criteria**

- Patient with an RIA previously treated endovascularly

- Patient with an allergic reaction to antiplatelets (tirofiban, aspirin, clopidogrel, prasugrel and cilostazol) or contrast

- Patient with a high risk of hemorrhage such intracerebral hemorrhage or severe gastric ulceration

- Patient with a high risk of strokes (over 50% cerebral artery stenosis or cerebral artery occlusion, moyamoya disease, atrial fibrillation)

- Patient with coagulopathy

- Patient with thrombocytopenia (thrombocytopenia (<100,000/mm^3^)

- Patient with liver diseases (>100IU/dL of aspartate aminotransferase or alanine aminotransferase)

- Patient with renal diseases (>2 mg/dL of serum creatinine)

- Patient with uncontrolled congestive heart failure or angina

- Patient with malignant tumors

- Pregnant or breast-feeding patient

- Patient who are determined to be disqualified by researchers

**9.3. Sample size calculation**

To our knowledge, no randomized controlled trial has been conducted to directly compare BPC and HEC in patients with an RIA to determine which method results in a lower recurrence rate. Based on the event rates of a previous subgroup analysis of the recently ruptured, medium-sized aneurysms in the HELPS trial, during the 15-18 months follow-up period, the any rate of recurrence of recently ruptured medium-sized aneurysms in the bare platinum coil (BPC) and second-generation HEC groups were 64.4% and 37.3%, respectively.^9^ On the basis of the recurrence rate in this study, we assumed that the primary outcome rate would be 64.4% in the BPC group and 37.3% in the HEC group (with a rate difference of 27.1%). The calculated sample size was 106 patients, which provided 80% power to detect this rate difference in the primary outcome at the 2-sided significance level of .05. After assuming a drop rate of 10%, we enrolled the study candidates, targeting 118 patients who underwent coil embolization for an RIA (59 in each group).

**10. Study Methods**

**10.1. Study design**

A prospective, randomized and controlled clinical trial

**10.2. Randomization, blinding, and interventions with study groups**

Patients with an RIA are assigned to the BPC or HEC groups in a 1:1 ratio with random number generated by a web site program ([www.randomizer.org](http://www.randomizer.org)).

**10.3. Study withdrawal**

- patient who are enrolled, but does not meet inclusion/exclusion criteria

- patient who are not treated with allocated coil embolization therapy

- patient who will be lost to follow-up before 12 months

**10.4. Data collection and Study Flow**

Baseline data (sex, age), smoking, alcohol intake, medication, and medical history, preprocedural findings (modified Fisher grade, Hunt and Hess grade), clinical status (pre & post-modified Rankin Scale [mRS] score) and aneurysmal data (aneurysm diameter, neck size, dome-to-neck ratio, aspect ratio, aneurysm volume, location) will be collected. Procedural data (treatment technique stent type, stent length, coil volume, coiling method, occlusion grade, procedural complications, CSF drainage method) are also collected. Aneurysm obliteration grades were classified as described by the Raymond-Roy occlusion classification: complete, residual neck, or residual aneurysm.^10^

|  | Inclusion visit  & procedure and hospitalization | Follow-up visit at 1 month | Follow-up visit at 3 months | Follow-up visit at 6 months | Follow-up visit at 12 months |
| --- | --- | --- | --- | --- | --- |
| Visits | V0-1* | V2 | V3 | V4 | V5 |
| Time in relation to the intervention | Day 1 | Day 1 ± 0.5 month | Day 3 ± 1 month | Day 6 ± 1 month | Day 12 ± 1 month |
| Informed consent | ● |  |  |  |  |
| Collection of demographic information | ● |  |  |  |  |
| Past medical history/family history | ● |  |  |  |  |
| Vital signs | ● | ● | ● | ● | ● |
| Electrocardiogram | ● |  |  |  |  |
| Laboratory tests | ● |  |  |  |  |
| Inclusion/exclusion criteria confirmation | ● |  |  |  |  |
| Endovascular treatment | ● |  |  |  |  |
| Modified Rankin Scale | ● | ● | ● | ● | ● |
| Radiologic evaluation | ● |  | ●^†^ | ●^‡^ | ●^§^ |
| Event evaluation | ● | ● | ● | ● | ● |
| Concomitant drug check | ● |  |  |  |  |
| * V0 and V1 coincide if a participant meets the inclusion/exclusion criteria  ^†^Radiological evaluation using simple X-ray  ^‡^Radiological evaluation using magnetic resonance angiography  ^§^Radiological evaluation using transfemoral cerebral angiography | | | | | |

**10.6. Study outcomes**

**10.6.1. Primary outcome**

- any recurrence defined as any progression on the Raymond-Roy occlusion classification class during 12-month follow-up period.^10^

* Roy-Raymond occlusion classification class: complete, residual neck, or residual aneurysm^10^

**
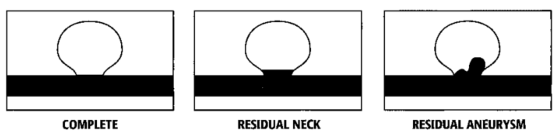
**

**10.6.2. Secondary outcomes**

1. Rate of retreatment during 12-month follow-up period.

2. Periprocedural complication

- any complication including thromboembolic events and hemorrhagic events during 1-month follow-up period

3. Delayed ischemic symptom during 12-month follow-up period

4. Good clinical outcome (mRS ≤ 2) at 12-month after coil embolization

5. rate of ventriculoperitoneal (VP) shunt operation during 12-month follow-up period

6. coil’s ease of manipulation

- Operator-perceived ease of coil manipulation was assesed using a 0-10 scale, with 0 representing extremely difficult handling and 10 representing extremely easy handling.

**10.7. Safety reporting**

: All expected and unexpected any clinical adverse event (AE) related with this study will be recorded in case report form (CRF).

: Serious adverse events (SAEs) will be reported to Institutional Review Board (IRB).

**10.7.1.AE**

: untoward medical occurrence with the use of an intervention in humans, whether or not considered intervention-related.

-> uncomfortable symptoms

-> physical signs

-> laboratory abnormalities

**10.7.2. SAEs**

: an AE or suspected AE is considered “serious” if, in the view of the investigator, it results in any of the following outcomes

-> death

-> a life-threatening adverse event

-> requiring hospitalization or prolongation of existing hospitalization

-> a persistent or significant incapacity or substantial disruption of the ability to conduct normal life functions

-> a congenital anomaly/birth defect

-> a medical event that may endanger the patient or require medical intervention to prevent one of above outcomes

**10.7.3. Suspected unexpected serious adverse reaction (SUSAR)**

: considered as SUSAR when it has unexpectedness and relationship to study intervention

**10.7.4. Reporting of SAEs and SUSAR.**

: SAEs

- SAEs should be reported to primary investigator within 24 hours after initial detection.

- SAEs will be collected and reported to individual IRB using a report form for safety data according to individual IRB SOP every 6 months.

: SUSAR

- SUSAR should be reported to primary investigator within 24 hours after initial detection.

- SUSAR will be reported to individual IRB using an AE report form according to individual IRB SOP within 15 days after initial detection

- In fatal or life-threatening event, SUSAR should be initially reported to IRB within 7 days after initial detection. Follow-up report will be added within 8 days thereafter.

**10.8. Statistical analysis**

**10.8.1. Timing of analyses**

: The primary outcome and its individual components will be analyzed on per-protocol (PP) basis at 12-month after randomization

: All secondary outcomes will be analyzed on PP basis at 12-month after randomization

**10.8.2. Analysis population**

: Per-protocol population

- Patients who maintain the initially assigned treatment group during 12-month follow-up period and completed follow-up throughout the designated study period.

**10.8.3. Statistical method**

Statistical analyses were performed using Stata statistical software, version 15 (StataCorp LP) and R version 4.2.2 (R foundation for Statistical Computing). Baseline demographics are presented as counts and percentage (categorical data) or mean and standard deviation (SD) for continuous data. Baseline characteristics were compared using the Wilcoxon rank sum test for continuous variables and the Chi-square or Fisher’s exact test for categorical variables, as appropriate. Multivariate logistic regression analyses will be performed to determine whether difference between the BPC and HEC groups existed for the following outcomes: 1) Any recurrence during the 12-month follow-up period, 2) retreatment during the 12-month follow-up period, and 3) favorable outcome (mRS ≤ 2) at 12-month follow-up. Multivariate logistic regression analyses will be adjusted for age, smoking status, aneurysm maximum diameter, aneurysm neck size, and the use of a stent. The findings were presented as coefficients, p-values, odds ratios (ORs), and 95% confidence intervals (CIs). Two-sided *P* values less than 0.05 were considered statistically significant.

**10.8.4. Interim analysis**

- Interim analysis will not be conducted.

**11. Data and safety committee (Data and Safety Monitoring plan)**

**11.1. Oversight responsibility**

Oversight of this study is provided by the principal investigator (O-Ki Kwon).

**11.2. Monitoring procedure**

- The principal investigator assures that informed consent is obtained prior to performing any research procedures, that all subjects meet eligibility criteria, and that the study is conducted according to the research plan. Study data are accessible at all times for the principal investigator to review. The principal investigator reviews study conducts on a monthly basis. The principal investigator reviews AEs individually real-time. The principal investigator reviews SAEs in real-time and in aggregate on 6-month basis.

**11.3. Reporting**

- The principal investigator ensures that all protocol deviations and SAEs are reported to IRB according to the applicable regulatory requirements

: minor protocol deviations -> every 1 year

: major protocol deviations -> within 15 days

: SAE -> every 6 months

: SUSAR (non-fatal/life-threatening) -> withing 15 days

: SUSAR (fatal/life-threatening) -> initial report within 7 days and follow-up report within 8 days thereafter

**12. Ethical considerations**

**12.1. Regulation statement**

This study will be started after approval by the IRB of the Seoul National University Bundang Hospital. The investigators will ensure that this study in conducted in full conformity with the Declaration of Helsinki (2013) and ICH-GCP guideline. The protocol, informed consent form, recruitment materials, and all participant materials will be submitted to the IRB for review and approval.

**12.2. Informed consent**

Informed consent forms will be IRB-approved and the participant will be asked to read and review the written document. The investigator will explain the research study to the participant and answer any questions that may arise. All participants will receive a verbal explanation in terms suited to their comprehension of the purpose, and potential risks of the study and of their rights as research participants. Language that the participant can understand will be provided. Participants will have the opportunity to carefully review the written consent form and ask questions prior to signing. The participants should have the opportunity to discuss the study with their surrogates or think about it prior to agreeing to participate. The participants will sign the informed consent document prior to any procedures being done specifically for the study. The participants can withdraw consent at any time throughout the course of the study. A copy of the written informed consent document will be given to the participants for their records. If the research participant does not fully understand the study, the study may proceed only if the participant's legally authorized representative has been provided with a full explanation of the study — using the same method as would be used for the participant — and gives consent after fully understanding it.

**12.3. Confidentiality Guarantee**

All names of participants shall be kept confidential, and participants shall be identified at the time of recording and evaluation by the number given in the clinical study. Inform the participant that all clinical trial data are stored on a computer and treated strictly confidentially. The signed automated clinical trial participant will be kept by the investigator. The consent of the clinical subject and the list of participants are kept for 3 years from the date of approval of the item.

**13. Store and Disposition of Human Derivative**

Not applicable

**14. References**

1. Molyneux AJ, Kerr RS, Clarke M, et al. International Subarachnoid Aneurysm Trial (ISAT) of neurosurgical clipping versus endovascular coiling in 2143 patients with ruptured intracranial aneurysms: a randomised comparison of effects on survival, dependency, seizures, rebleeding, subgroups, and aneurysm occlusion. *Lancet* 2005;366:809-817

2. Sadato A, Hayakawa M, Adachi K, et al. Large Residual Volume, Not Low Packing Density, Is the Most Influential Risk Factor for Recanalization after Coil Embolization of Cerebral Aneurysms. PLoS One 2016;11:e0155062

3. Bendok BR, Abi-Aad KR, Ward JD, et al. The Hydrogel Endovascular Aneurysm Treatment Trial (HEAT): A Randomized Controlled Trial of the Second-Generation Hydrogel Coil. Neurosurgery. 2020;86:615-624.

4. Imamura H, Sakai N, Sakai C, et al. Hydrogel coils in intracranial aneurysm treatment: a multicenter, prospective, randomized open-label trial. J Neurosurg. 2025;142:1803-1809.

5. Taschner CA, Chapot R, Costalat V, et al. Second-Generation Hydrogel Coils for the Endovascular Treatment of Intracranial Aneurysms: A Randomized Controlled Trial. Stroke. 2018;49:667-674.

6 White PM, Lewis SC, Gholkar A, et al. Hydrogel-coated coils versus bare platinum coils for the endovascular treatment of intracranial aneurysms (HELPS): a randomised controlled trial. The Lancet. 2011;377:1655-1662.

7. Corns R, Zebian B, Tait MJ, et al. [Prevalence of recurrence and retreatment of ruptured intracranial aneurysms treated with endovascular coil occlusion.](https://pubmed.ncbi.nlm.nih.gov/22762269/) Br J Neurosurg. 2013;27:30-33.

8. Rezek I, Mousan G, Wang Z, Murad MH, Kallmes DF. Coil type does not affect angiographic follow-up outcomes of cerebral aneurysm coiling: a systematic review and meta-analysis. AJNR Am J Neuroradiol. 2013;34:1769-73

9. Brinjikji W, White PM, Nahser H, et al. HydroCoils reduce recurrence rates in recently ruptured medium-sized intracranial aneurysms: a subgroup analysis of the HELPS trial. AJNR Am J Neuroradiol. 2015;36:1136-1141.

10. Roy D, Milot G, Raymond J : Endovascular treatment of unruptured aneurysms. Stroke 2001;32:1998-2004

11. Meyers PM, Schumacher HC, Higashida RT, et al. Reporting standards for endovascular repair of saccular intracranial cerebral aneurysms. Stroke 2009;40:e366-79.

**Supplementary Method 3** Inclusion and Exclusion Criteria

**Inclusion criteria**

- Patients with a ruptured intracranial aneurysm (RIA) who plan to undergo coil embolization

- Patient aged ≥19 years

- Patient who agrees to this study (with informed consent)

**Exclusion criteria**

- Patient with an RIA previously treated endovascularly

- Patient with an allergic reaction to antiplatelets (tirofiban, aspirin, clopidogrel, prasugrel and cilostazol) or contrast

- Patient with a high risk of hemorrhage such intracerebral hemorrhage or severe gastric ulceration

- Patient with a high risk of strokes (over 50% cerebral artery stenosis or cerebral artery occlusion, moyamoya disease, atrial fibrillation)

- Patient with coagulopathy

- Patient with thrombocytopenia (thrombocytopenia (<100,000/mm^3^)

- Patient with liver diseases (>100IU/dL of aspartate aminotransferase or alanine aminotransferase)

- Patient with renal diseases (>2 mg/dL of serum creatinine)

- Patient with uncontrolled congestive heart failure or angina

- Patient with malignant tumors

- Pregnant or breast-feeding patient

- Patient who are determined to be disqualified by researchers

**Supplementary Result 1** Overall clinical outcomes

|  | Patients, No. (%) | | | |
| --- | --- | --- | --- | --- |
| Characteristics | Overall  (n = 57) | BPC  (n = 26) | HEC  (n = 31) | P value |
| Preprocedural mRS |  |  |  |  |
| 0 | 1 (1.8) | 0 (0.0) | 1 (3.2) | 0.338 |
| 1 | 30 (52.6) | 13 (50.0) | 17 (54.8) |  |
| 2 | 12 (21.1) | 5 (19.2) | 7 (22.6) |  |
| 3 | 6 (10.5) | 4 (15.4) | 2 (6.5) |  |
| 4 | 6 (10.5) | 4 (15.4) | 2 (6.5) |  |
| 5 | 2 (3.5) | 0 (0.0) | 2 (6.5) |  |
| Final follow-up mRS |  |  |  | 0.979 |
| 0 | 44 (77.2) | 19 (73.1) | 25 (80.6) |  |
| 1 | 3 (5.3) | 2 (7.7) | 1 (3.2) |  |
| 2 | 4 (7.0) | 2 (7.7) | 2 (6.5) |  |
| 3 | 2 (3.5) | 1 (3.8) | 1 (3.2) |  |
| 4 | 2 (3.5) | 1 (3.8) | 1 (3.2) |  |
| 5 | 2 (3.5) | 1 (3.8) | 1 (3.2) |  |
| *BPC* bare platinum coil, *HEC* hydrogel embolic coil, *mRS* modified Rankin Scale | | | | |

**Supplementary Result 2** Sensitivity Analysis of primary and secondary outcomes (‘worst-case’ scenario)

|  | No. (%) | | | |
| --- | --- | --- | --- | --- |
|  | Overall (n = 68) | BPC (n = 32) | HEC (n = 36) | P value^*^ |
| Primary outcome |  |  |  |  |
| Any recurrence^†^ | 27 (39.7) | 16 (50.0) | 11 (30.6) | 0.138 |
| Secondary outcome |  |  |  |  |
| Retreatment | 17 (25.0) | 9 (28.1) | 8 (22.2) | 0.589 |
| Procedural complication | 12 (17.6) | 6 (18.8) | 6 (16.7) | >0.999 |
| Delayed cerebral ischemia | 13 (19.1) | 8 (25.0) | 5 (13.9) | 0.356 |
| Favorable clinical outcome (mRS ≤ 2) | 51 (75.0) | 23 (71.9) | 28 (77.8) | 0.589 |
| CSF drainage^‡^ | 39 (57.4) | 21 (65.6) | 18 (50.0) | 0.226 |
| VP shunt operation | 16 (23.5) | 10 (31.2) | 6 (16.7) | 0.252 |
| *BPC* bare platinum coil, *HEC* hydrogel embolic coil, *CSF* cerebrospinal fluid, *VP* ventriculoperitoneal, *mRS* modified Rankin Scale, *SD* standard deviation  *The P value measured the comparison between the BPC group and HEC group.  ^†^Any recurrence defined as any progression in the Raymond-Roy occlusion classification class during the 12-month follow-up period.  ^‡^Cases in which external ventricular drainage catheter insertion or lumbar drainage catheter insertion was performed either before or after the procedure are included. | | | | |

**Supplementary Result 3** Sensitivity Analysis of primary and secondary outcomes (‘best-case’ scenario)

|  | No. (%) | | | |
| --- | --- | --- | --- | --- |
|  | Overall (n = 68) | BPC (n = 32) | HEC (n = 36) | P value^*^ |
| Primary outcome |  |  |  |  |
| Any recurrence^†^ | 16 (23.5) | 10 (31.2) | 6 (16.7) | 0.252 |
| Secondary outcome |  |  |  |  |
| Retreatment | 6 (8.8) | 3 (9.4) | 3 (8.3) | >0.999 |
| Procedural complication | 1 (1.5) | 0 (0.0) | 1 (2.8) | >0.999 |
| Delayed cerebral ischemia | 2 (2.9) | 2 (6.2) | 0 (0.0) | 0.218 |
| Favorable clinical outcome (mRS ≤ 2) | 62 (91.2) | 29 (90.6) | 33 (91.7) | >0.999 |
| CSF drainage^‡^ | 28 (41.2) | 15 (46.9) | 13 (36.1) | 0.461 |
| VP shunt operation | 5 (7.4) | 4 (12.5) | 1 (2.8) | 0.180 |
| *BPC* bare platinum coil, *HEC* hydrogel embolic coil, *CSF* cerebrospinal fluid, *VP* ventriculoperitoneal, *mRS* modified Rankin Scale, *SD* standard deviation  *The P value measured the comparison between the BPC group and HEC group.  ^†^Any recurrence defined as any progression in the Raymond-Roy occlusion classification class during the 12-month follow-up period.  ^‡^Cases in which external ventricular drainage catheter insertion or lumbar drainage catheter insertion was performed either before or after the procedure are included. | | | | |
